# Superresolved polarization-enhanced second-harmonic generation for direct imaging of nanoscale changes in collagen architecture

**DOI:** 10.1364/OPTICA.411325

**Published:** 2021-05-13

**Authors:** Peter B. Johnson, Artemios Karvounis, H. Johnson Singh, Christopher J. Brereton, Konstantinos N. Bourdakos, Kerry Lunn, James J. W. Roberts, Donna E. Davies, Otto L. Muskens, Mark G. Jones, Sumeet Mahajan

**Affiliations:** 1 School of Chemistry, Faculty of Engineering and Physical Sciences, University of Southampton, Southampton, UK; 2 Institute for Life Sciences, University of Southampton, Southampton, UK; 3 Optoelectronics Research Centre and Centre for Photonic Metamaterials, University of Southampton, Southampton, UK; 4 Physics and Astronomy, Faculty of Engineering and Physical Sciences, University of Southampton, Southampton, UK; 5 NIHR Southampton Biomedical Research Centre, University Hospitals Southampton, Clinical and Experimental Sciences, Faculty of Medicine, University of Southampton, Southampton, UK; 6 Synairgen Research Ltd., Southampton, UK

## Abstract

Superresolution (SR) optical microscopy has allowed the investigation of many biological structures below the diffraction limit; however, most of the techniques are hampered by the need for fluorescent labels. Nonlinear label-free techniques such as second-harmonic generation (SHG) provide structurally specific contrast without the addition of exogenous labels, allowing observation of unperturbed biological systems. We use the photonic nanojet (PNJ) phenomena to achieve SR-SHG. A resolution of ∼λ/6 with respect to the fundamental wavelength, that is, a ∼2.3-fold improvement over conventional or diffraction-limited SHG under the same imaging conditions is achieved. Crucially we find that the polarization properties of excitation are maintained in a PNJ. This is observed in experiment and simulations. This may have widespread implications to increase sensitivity by detection of polarization-resolved SHG by observing anisotropy in signals. These new, to the best of our knowledge, findings allowed us to visualize biological SHG-active structures such as collagen at an unprecedented and previously unresolvable spatial scale. Moreover, we demonstrate that the use of an array of self-assembled high-index spheres overcomes the issue of a limited field of view for such a method, allowing PNJ-assisted SR-SHG to be used over a large area. Dysregulation of collagen at the nanoscale occurs in many diseases and is an underlying cause in diseases such as lung fibrosis. Here we demonstrate that pSR-SHG allows unprecedented observation of changes at the nanoscale that are invisible by conventional diffraction-limited SHG imaging. The ability to nondestructively image SHG-active biological structures without labels at the nanoscale with a relatively simple optical method heralds the promise of a new tool to understand biological phenomena and drive drug discovery.

## 1. INTRODUCTION

The Abbe “diffraction limit” states that the resolution of an optical microscope is limited by its wavelength to ∼λ/(2NA), with NA being the numerical aperture of the imaging system. In biological microscopy, this poses a major limitation, as a wealth of processes occur on a scale below the diffraction limit. Many techniques now exist to surpass this limit, both in the far field: stimulated emission depletion (STED) microscopy, localization microscopies such as PALM/STORM, structured illumination microscopy (SIM) and in the near field: near-field scanning optical microscopy (NSOM) [1,2], and solid immersion microscopy [3], collectively referred to as superresolution (SR) techniques [4]. However, these techniques are either complex to implement or rely on the properties of

**Fig. 1.**
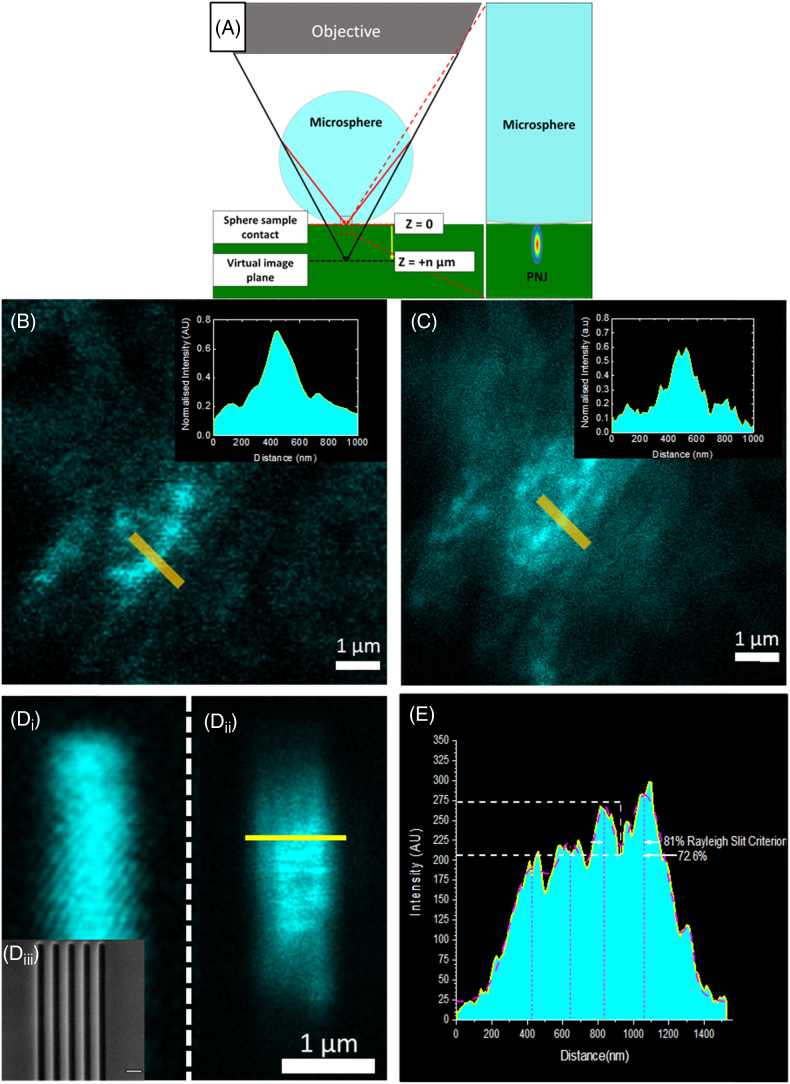
Establishing the limit of resolution of SR-SHG. (A) Diagram depicting imaging using PNJs generated using a microsphere in a microscope setup. The microsphere is located on the surface of the sample and acts to focus the light to a subdiffraction-limited spot. By scanning the illumination laser beam, the PNJ is scanned on the sample, forming an SR virtual image at distance z on the shadow side of the sphere. (B) Diffraction-limited SHG signal from a fibrotic lung tissue sample containing collagen; (C) SHG image of the same region taken using a PNJ. It is possible to resolve more fibers and their directionality. Insets show intensity plots taken over the yellow lines in (B)–(C). More peaks are resolved in the PNJ image compared to the diffraction-limited image. ($D_{iii}$) SEM image of the silicon sample; each dark slit is 100 nm wide and the slits are separated by 125 nm; ($D_{i}$) diffraction-limited image of the silicon slit sample; the subdiffraction-limited features are resolved. The reduced FOV of the high resolution microsphere is apparent. ($D_{ii}$) Image taken through a 14 µm sphere of the silicon slit sample; the subdiffraction-limited features are resolved. (E) Intensity plot taken across the yellow line in $D_{ii}$. The plot shows four peaks corresponding to slits; the peaks can be resolved with a drop to 72.6% intensity, satisfying the Rayleigh criterion.

fluorescent labels, or both. As such, achieving SR using label-free techniques such as second-harmonic generation (SHG) microscopy would be of great interest, but so far this modality has not seen the same success. SHG microscopy is a structurally specific technique that highlights noncentrosymmetric features without the addition of exogenous and potentially perturbative labels. SHG active materials include certain crystals such as barium titanate ($BaTiO_{3}$) and biological macromolecules including the actin-myosin complex, microtubules, and fibrillar collagen. The ability to perform far-field label-free SHG imaging with subdiffraction-limited resolution therefore holds significant potential for biomedical research, as changes in collagen structure and function underlie many diseases including fibrosis and cancers.

To realize label-free SR-SHG imaging, we have employed the photonic nanojet (PNJ) phenomena, as it does not rely on fluorescent switching/blinking and is simple to implement. A PNJ is a localized focus of light with an intensity cross section narrower than the diffraction limit that is able to propagate over multiple wavelengths [5]. Initial demonstrations of the phenomena used high refractive index spheres or cylinders to generate the PNJ, and this is still the most common experimental implementation; however, theoretical studies have investigated cuboids [6], cones, prism structures [7], and spheres with multiple/graded refractive indices [8]. Typically, dielectric spheres of sizes between 5 and 100 µm in diameter are placed on the surface of a sample to focus the light into a PNJ, decreasing the illumination point spread function (PSF) of the system [9]. By scanning the illuminating beam over the sphere, the PNJ is scanned over the sample, forming a virtual image that is detected in the far field using the illumination objective (Fig. 1(A)). Whispering gallery modes [10], elements of solid immersion lens theory [11,12] and near-field effects [13–15] have also been suggested as contributing to the SR imaging capability of PNJ-generating microspheres.

The focal spot of a PNJ, when generated using a microsphere, is dependent on multiple parameters, including sphere size, imaging wavelength, and the refractive index contrast with the surrounding medium [16]. For biological imaging, the contrast has to be ideally maximized against the microscope immersion medium. Imaging with PNJs (Fig. 1(A)) has been typically performed in a wide-field configuration, using both bright field and epifluorescence [17,18]. It has also been demonstrated in a laser-scanning configuration [19] and is amenable to SHG microscopy [20].

SHG interrogates the second-order polarizability of materials and detects noncentrosymmetric structures. It is widely used to image fibrillar collagen in bioimaging [21]. SHG is polarization-sensitive; intensities depend on the relative orientation between the excitation and the SH active structure. Thus, polarization-resolved SHG (p-SHG) is used to acquire information about the orientation and degree of organization of harmonophores, for example, to calculate the helical pitch angle of collagen fibrils [22] and the tilt angle of the helices relative to the fibril axis [23]. It has also been used to understand the differences between collagen types in tissues [24] and investigate the effect of mechanical stretching [25] on collagen disorder within tendon tissue. Collagen and its deposition/accumulation plays a critical role in many diseases, especially fibrotic diseases such as liver fibrosis and idiopathic lung fibrosis [26–28]. The ability to resolve SHG-active structures at the nanoscale is vital as changes occur at the level of molecular structures and assemblies such as the actin-myosin complex [29], microtubules [30], and fibrillar collagen [31–33], all of which are SHG-active. SHG has already proved to be a valuable tool for biomedical and biophysical investigations; however, subdiffraction-limited resolution has not been achieved, preventing nanoscopic analysis.

Here we present the first demonstration of SR-SHG microscopy. We use PNJs generated using a microsphere to achieve SR-SHG imaging in the far field and demonstrate a route to extended field of view of superresolved SHG imaging. We investigate the origin of SR imaging using PNJs highlighting a critical near-field component. Most importantly, we find that polarization properties of the excitation are maintained in a PNJ. This unexpected finding crucially enabled polarization-resolved SR-SHG (pSR-SHG), which further enhances resolution. In an unprecedented and highly relevant application, we show that nanoscopic anisotropy analysis enabled by pSR-SHG allows the detection of underlying nanoscale changes in collagen during the development of lung fibrosis. Overall, our work heralds the role that label-free SR techniques can play to unravel underlying biological phenomena and improve our understanding of disease mechanisms.

## 2. RESULTS AND DISCUSSION

Achieving SR-SHG imaging can have applications in understanding nanoscale biology and evolution of SHG-active materials, such as in disease processes. Here, we used the PNJ phenomena to achieve SR-SHG (Fig. 1(A)). The SHG signals observed by excitation via a PNJ showed the expected second-order dependence on power on imaging barium titanate nanocrystals (Supplement 1, Fig. S1A and S1B). In the context of SHG from single collagen fibers, it has been shown that with higher excitation power, sensitivity to smaller fiber diameters is achieved [34]. We clearly also see that at higher power, the signal-to-noise ratio (SNR) improves in PNJ-assisted SHG (Supplement 1, Fig. S1(C–E)). Since SNR is inherently linked to resolution, further experiments were conducted at the highest excitation power possible (typically ∼30 mW) within the damage threshold of the sample to obtain the highest resolution within the imaging conditions.

We acquired images of the same location of the sample without and with PNJ excitation of SHG to investigate the extent of improvement over conventional diffraction-limited SHG. A clear improvement in resolution is apparent in SHG signals from barium titanate nanocrystals (Supplement 1, Fig. S2) and a lung tissue section (Figs. 1(B) and 1(C)), which was verified to have an abundance of fibrillar collagen (Supplement 1, Fig. S3). We demonstrated that PNJ-assisted SHG imaging allowed the distinction of individual fibers and their directionality, an observation that was not possible with conventional diffraction-limited imaging. A typical collagen fiber is between 12 and 500 nm (age-dependent) in diameter, as determined previously by electron microscopy [35]. The diffraction-limited resolution of our SHG microscope at the fundamental wavelength (800 nm) is ∼288nm (0.61λ/√2N.A) [36], whereas the ultimate diffraction limit in an aqueous medium is ∼259nm. Hence, the ability to resolve fibers at this separation suggests the SR capability of PNJ-excited SHG.

In order to quantitatively determine the resolution limit for SR-SHG, a patterned silicon sample was fabricated of five individual cuts etched on a thin silicon membrane using a focused ion beam. The width of each cut is 100 nm, while the spacing between them is 125 nm (λ/6.4) verified by transmission electron microscopy (TEM) (Fig. 1(D)(iii)). Figure 1(D)(i) shows an image of the silicon sample acquired using diffraction-limited SHG; Fig. 1(D)(ii) shows the same field of view (FOV) acquired through a 14 µm diameter sphere to generate a PNJ. The relationship between size and magnification is shown in Supplement 1, Fig. S4; smaller spheres can achieve better resolution due to their narrower PNJ [9]. As shown in Fig. 1(E) (Supplement 1, Fig. S5) the slits can be well resolved, satisfying the Rayleigh criterion for slits (81% of peak) [37,38]. This shows that SR-SHG can be achieved with at least 125 nm resolution, which is 1.68 times better than anything achieved in SHG microscopy [39]. In our experiments, the limitation was due to lack of available SHG-active standards and the fabricated sample exploited surface SHG that is significantly weaker than bulk SHG [40]. This could not be surmounted by applying higher laser power, given the damage threshold for such thin samples. Hence, the actual achievable resolution with our PNJ-assisted SR-SHG method may be even higher.

**Fig. 2.**
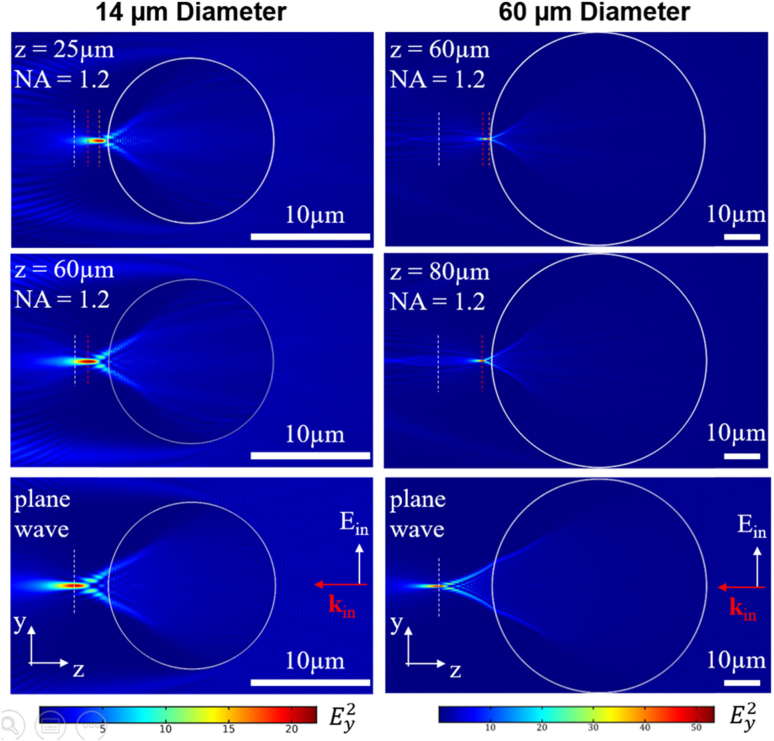
Simulations (2D) of PNJ formation under plane wave versus Gaussian illumination using an NA of 1.2 for 14 µm diameter (left column) and 60 µm diameter (right column) spheres. Color maps indicate field intensity in the y-polarization component $E_{y}^{2}$ for different illumination conditions corresponding to plane-wave illumination (bottom) and a focused Gaussian beam with NA=1.2. Different distances of the illumination focus from the sphere end surface, z, are shown in middle and top rows, as indicated in the figure. Colored dashed lines represent the position of the maximum intensity of the PNJ for the three conditions for each sphere. Scale bars, 10 µm.

**Fig. 3.**
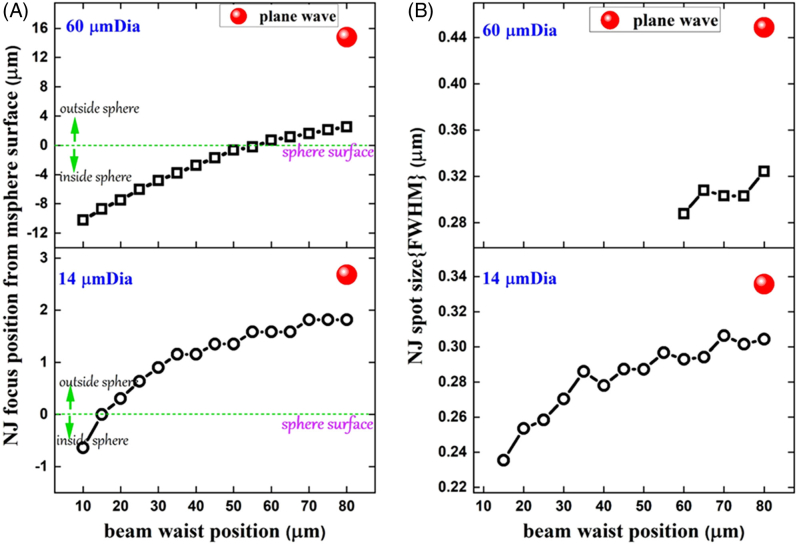
Analysis of simulations of nanojet formation under Gaussian illumination. (A) Nanojet focus position as a function of illuminating beam waist position. For a 60 µm diameter sphere, the nanojet first forms outside of the microsphere when the illuminating beam is focused 60 µm from the sphere surface; as the illumination focus is moved farther from the sphere the nanojet focus also moves farther from the sphere surface. The same trend is true for smaller spheres; however, the nanojet focus first forms outside of the sphere when the illumination focus is ∼15µm below the sphere surface. (B) Nanojet spot size as a function of illumination focus position. The nanojet produced by both sizes of the sphere is narrowest when the illuminating beam is focused close to the microsphere surface. The narrowest achievable spots are 285 and 235 nm for 60 and 14 µm diameter spheres, respectively. Spots of a narrower width than this form inside of the microsphere and therefore cannot be used for imaging.

Despite imaging occurring in the far field, it has been proposed that the SR effect of PNJs has a strong near-field component [41,42]. To further understand this phenomenon, simulations were performed for both 14 and 60 µm diameter spheres using Gaussian beam illumination with a numerical aperture (NA) of 1.2 as used in experiments. The PNJ is observed both under plane-wave and focused-illumination conditions (Fig. 2); however, it is seen that the PNJ maximum moves much closer to the surface of the sphere under focused Gaussian illumination (Fig. 3(A)). This behavior can be understood as the effect of a compound lens consisting of both microscope objective and the microsphere where the effective focus is shifted towards the sphere. Here the distance of the sphere with respect to the focus of the microscope is a critical parameter, z representing the distance of the microsphere end surface from the objective focus position in absence of the microsphere.

**Fig. 4.**
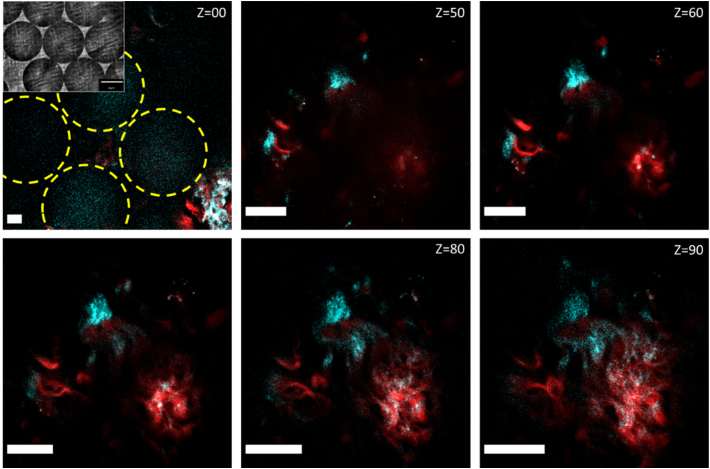
Arrayed spheres allow simultaneous acquisition of multiple images. Images shown of a group of spheres deposited on a lung tissue sample; cyan signal is SHG; red signal is two-photon excited fluorescence. In the Z=00 image, the locations of four neighboring spheres can be seen indicated by yellow circles; three of these contribute to clear FOVs when imaging through the spheres. As the focal position is changed (Z is increased), the magnification increases, as does the resolution. At Z=70µm, magnification is maximized while ensuring nonoverlapping FOVs. At greater values of Z, overlap confounds image information from each sphere. Inset, representative bright-field image showing six spheres packed together. Scale bars, 10 µm.

As the PNJ moves closer to the sphere surface, the components of near-field components increase, resulting in a reduced spot size (Fig. 3(B)). The smallest achievable single-photon PNJ waist sizes in our simulations are 285 and 235 nm for the 60 and 14 µm diameter spheres, respectively, due to the square-law dependence of SHG the size of the effective PSF will be reduced by a factor of √2, giving approximate SHG spot sizes of 201 and 166 nm, respectively. The diffraction-limited SHG resolution for a 1.2 NA objective is 288 nm, and therefore the achieved spot sizes from simulations are well below the diffraction limit for this system and provide an explanation for the improved resolution observed in the experiments. This is in line with previously published results [9].

While SR is clearly demonstrated with PNJ-assisted SHG, a potential limitation is the restriction of the FOV given by one sphere. Figure 1(D) shows four distinguishable stripes due to the location of the sphere on the sample, as the FOV is limited by the sphere size. We applied a simple multiplexing technique to allow rapid acquisition of multiple SR-SHG images using self-assembled ordered arrays of spheres (Fig. 4, inset) to overcome this limitation. The magnification achieved is dependent on the size of the sphere used and the focal plane z position (Supplement 1, Fig. S4). Therefore, if taken with an array of spheres, the image from neighboring spheres can overlap and hence needs to be optimized. With a 60 µm diameter sphere, a focal position of z=70µm was found to be the optimum to maximize resolution while avoiding overlap of images (Fig. 4). The close packing of spheres allows for maximal effective imaging FOV without moving the sphere array. The FOV of a single sphere is smaller than the diameter of the sphere (⊘∼15µm for ⊘=60µm sphere); thus, there are unimaged portions between spheres. Although not demonstrated for SR-SHG, multiple spheres and image stitching have been used to reconstruct composite FOVs [43–45] and can be applied to our approach. Our approach retains the advantage of simplicity while offering increased speed of data acquisition relative to single-sphere approaches, and most importantly, is demonstrated for label-free laser-scanning nanoscopy (specifically SR-SHG).

**Fig. 5.**
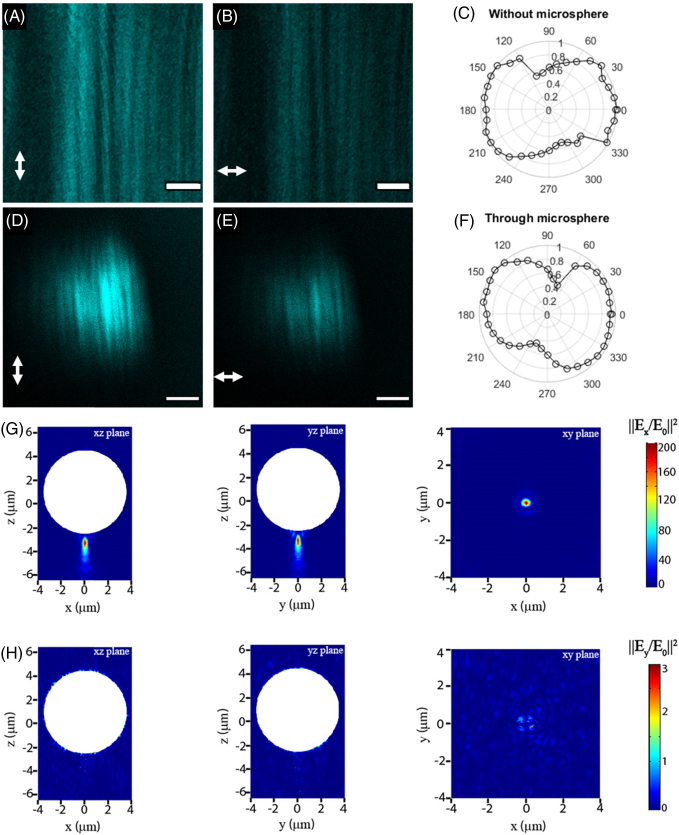
PNJs maintain the polarization of light. (A) and (B) Images of collagen fibers in mouse tail tendon with the excitation polarization aligned parallel and perpendicular to the fiber orientation, respectively; (D) and (E) same situation as (A) and (B) with imaging performed using PNJs. In both cases, the intensity is at a maximum when fibers and excitation polarization are aligned and at a minimum when they are perpendicular. (C) and (F) show polar plots of signal intensity against excitation polarization for the diffraction-limited and PNJ cases, respectively. The shape of the plots is the same, showing that polarization is maintained through microspheres. Scale bars, 3 µm. (G) Simulation results, color map plot of the x component of the electric field intensity $\left({||E/E_{0}||}^{2}\right)$ in various planes. The incident light was x-polarized. The white shaded circular region represents the microsphere. The xz and yz correspond to planes passing through the center of the microsphere, while the xy plane is plotted passing through the focal point of the generated PNJ. (H) Same as (G), but for the y component. The intensity of the y component is approximately 2 orders of magnitude smaller than the x component, indicating that the polarization state of the illumination is not significantly affected when focused onto a PNJ.

Having shown that SR-SHG is possible using the PNJ approach and that the FOV can be expanded, we sought to investigate ways to achieve ultrahigh resolution and develop a technique that can be used for distinction at the nanoscale level. Hence, we examined the property that in SHG, signals are dependent on the relative orientation of harmonophores versus the polarization of the excitation [46]. Furthermore, the relative orientation of neighboring harmonophores both to each other and to the excitation heavily influence the ability to resolve these structures. We conducted experiments with different polarizations with a multiple slit sample, the same as that shown in Fig. 1. The results show that with alignment of orientation, the slits are resolved (Supplement 1, Fig. S6C). A similar result is obtained with two closely spaced barium titanate crystals (Supplement 1, Figs. S6D and S6E). Moreover, this dependence has been used previously to achieve SR using SHG labels [47]. To demonstrate this sensitivity to polarization, we used a mouse tail tendon that contains a high density of aligned collagen fibers (Fig. 5). The polar plots (Figs. 5(C) and 5(F)) showed the dumbbell shape, as expected for polarization-dependent SHG signals [48]. However, this strong polarization-dependent behavior of SHG observed even through a microsphere was intriguing. The experimental result suggests that polarization is maintained despite complex propagation conditions (whispering gallery modes, near fields, etc.) that are taking place in the microspheres and that are responsible for the tight focusing [2–9]. This was unexpected, and hence, to verify this result, we performed three-dimensional (3D) numerical simulations, and the polarization state of the PNJ of a microsphere was investigated. Figures 5(G) and 5(H) show the x and y components of the electric field ($\|E/E_{0}{\|}^{2}$), respectively, when the sphere is illuminated with linearly polarized light in the x plane. The intensity of the y component in the PNJ is approximately 2 orders of magnitude smaller than the x component, indicating that the polarization state of the illumination is not significantly affected when focused into a PNJ. These results indicate that it should be possible to perform SR p-SHG measurements.

**Fig. 6.**
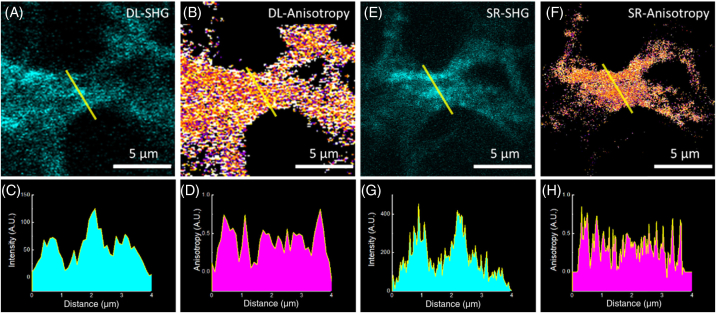
Polarization anisotropy reveals features that cannot be identified by intensity alone. Diffraction-limited SHG (A) intensity and (B) anisotropy images of collagen in a lung cell spheroid; (C) an intensity plot taken through the yellow line in A shows clear peaks from collagen fibrils. The corresponding anisotropy measurement (D) reveals new features at finer spatial resolution than the intensity alone. (E) SR-SHG measurement of the same FOV as (A) reveals many finer features (G). SR anisotropy measurements (F) show that PNJs allow greater resolution of changes in anisotropy (H).

Polarization-dependent changes can be measured by polarization anisotropy; $r=(I_{∥}-I_{⊥})/\;(I_{∥}+2I_{⊥})$, where $I_{∥}$ and $I_{⊥}$ are the intensities recorded parallel or perpendicular to incident polarization. Polarization anisotropy can vary between −0.5 and 1, where 0 is completely random emission and 1 is emission completely aligned with the excitation polarization. Pixel-wise calculation of anisotropy was performed to generate images (Fig. 6(B)/6(F)) that show the spatial distribution of disorder within the collagen ultrastructure. The changes in disorder can occur at a subfiber scale; thus, it is possible to observe changes that are not detectable using intensity alone; different features showing higher frequency changes can be seen in collagen fibers on using polarization anisotropy on the same sample (Supplement 1, Fig. S7). The same location taken from a sample containing collagen fibrils (lung cell culture model; see Materials and Methods) was imaged. Figures 6(A) and 6(B) show the diffraction-limited, while Figs. 6(E) and 6(F) show the PNJ-assisted SHG intensity and corresponding polarization anisotropy images. Line profile plots show an increased number of peaks and reveal hidden features in PNJ-assisted SHG imaging. The narrower width of the PNJ versus the diffraction-limited PSF allows the resolution of both finer fiber features via intensity (Fig. 6(C) versus Fig. 6(G)) and further identification of subfiber changes via anisotropy (Fig. 6(D) versus Fig. 6(H)). This demonstrates that polarization-resolved SHG, herein using the anisotropy, offers sensitivity to SR structural changes; we term these sets of techniques as pSR-SHG. Polarization has been used previously to improve the resolution of diffraction-limited fluorescence images [49,50] by improving the localisation accuracy of the dye molecules used for labeling biological samples. Here, however, we crucially show that polarization anisotropy not only increases resolution of SHG, but also allows resolution of neighboring nanoscale collagen fibrils, improving both the sensitivity and the resolution even in PNJ-assisted (hence, superresolved) SHG measurements while providing additional structural information and maintaining the benefits of label-free imaging. The anisotropy of polarization-sensitive SHG diffraction-limited imaging itself is powerful and can be used to delineate different stages of ovarian cancer [51] and differentiate between different collagen isoforms [52]. However, in many cases, the changes are much more subtle and occur at the nanoscale, such as in idiopathic lung fibrosis [53]. Here we applied our simple superresolved SHG imaging approach, particularly pSR-SHG, to see whether such nanoscale changes in collagen could be observed directly.

**Fig. 7.**
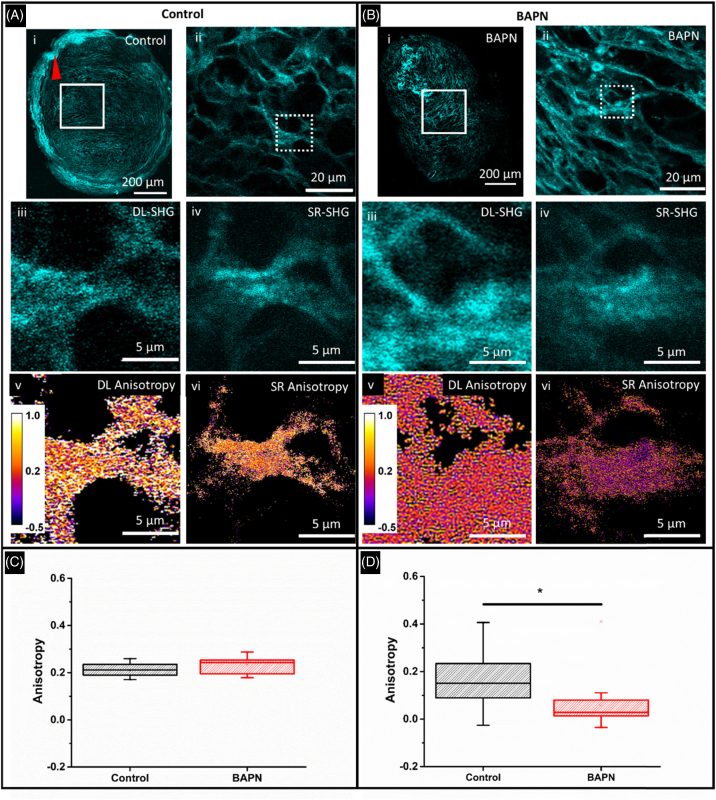
SR-SHG polarisation anisotropy measurements reveal nanoscale differences in collagen ultra-structure under treatment with LOX/LOXL inhibitors. SHG imaging of lung spheroids shows differing collagen morphology at the edge of the spheroid [loss of ordered collagen indicated by red arrowhead (Ai) versus (Bi)]. (A/Bii) Zoomed view of white boxed region in (A/Bi). Similar collagen morphology occurs within the central region of the spheroid between control and treatment with LOX/LOXL inhibitor BAPN. When observed at higher magnification [dashed squares in (A/Bii) using either diffraction-limited (A/Biii) or SR-SHG (A/Biv)], the collagen morphology remains similar. Diffraction-limited anisotropy images show that the measured anisotropy is unchanged between the treatments (Av) versus (Bv), which is quantified in (C) mean anisotropies are 0.2132 and 0.2322 for control and BAPN-treated, respectively (15 FOVs for control and BAPN). The SR anisotropy images (A/Bvi) and measurements (D) highlight a significant difference in the mean anisotropy. Control, 0.1471 (29 spheres) BAPN, 0.0600 (20 spheres). BAPN reduces the anisotropy. Significance tested using a one way ANOVA, P=0.0131.

Diffraction-limited as well as PNJ-assisted SHG images of sections from a tissue spheroid model of the fibrotic lung disease, idiopathic pulmonary fibrosis (IPF) were acquired (Fig. 7). We have previously shown, using TEM (Supplement 1, Fig. S8) that in this model, the collagen ultrastructure changes significantly following treatment with lysyl-oxidase/lysyl-oxidase-like (LOX/LOXL) inhibitor b-aminoproprionitrile (BAPN), which inhibits cross-linking of collagen fibers [53]. Figure 7 shows diffraction-limited SHG images for both the control (A) and BAPN-treated (B) sections. We have previously demonstrated that the macroscopic collagen architecture becomes more disordered upon treatment with BAPN [53], and here this can be seen by the loss of the high-intensity collagen ring at the edge of the sphere (Fig. 7(A)(i), red arrow). However, the central regions of the spheroids show very similar collagen architecture when viewed at the diffraction limit, which was further verified via image analysis of fibers using a number of parameters such as fiber length and width (Supplement 1, Fig. S9). The polarization anisotropy measured from diffraction-limited images is unchanged (Fig. 7(C)). SR-SHG images taken of the same spheroid samples also show that the collagen architecture is similar between the control and on BAPN treatment (Fig. 7(A)/7(B)(iv), Supplement 1, Fig. S9). However, SR-SHG indicated that the mean fiber widths for control and BAPN-treated samples were 119 and 158 nm, respectively. This increase is in agreement with electron microscopy analysis (Supplement 1, Fig. S8). These widths are much narrower than the ∼550nm widths measured by diffraction-limited imaging. We propose that this is due to the measurement of bundled fibers that cannot be resolved at the diffraction limit but can be resolved using PNJs. SR-SHG analysis also showed shorter fiber lengths (1–3 µm) for both control and BAPN-treated spheroids than diffraction-limited imaging (>3µm). The range of fiber lengths is above the diffraction-limited resolution of our system (288 nm), and therefore the shorter fibers shown by SR-SHG is intriguing. It has been shown that the segmented fiber structure observed in epidetected SHG is a result of the fibril size and their packing density in the axial direction [54]. The axial extent of the PNJ is 1.4 µm (Supplement 1, Fig. S9), ∼2× as long as the diffraction-limited PSF of 0.657 µm calculated based on [55]. Thus, the PNJ method can probe a larger axial distribution of SHG harmonophores, giving rise to different observed fiber lengths due to increased sensitivity. Furthermore, pSR-SHG shows that there is a significant difference between the control and BAPN-treated sample (Fig. 7(D)). This is consistent with our previous observation using TEM of dysregulation of collagen fibril nanostructure throughout the spheroid model [53]. We attribute the improved sensitivity to changes in collagen ultrastructure to the tight focusing of the PNJ. The subdiffraction-limited width of the PNJ allows detection of changes in anisotropy on a finer spatial scale (Fig. 6(H)). In addition, the PNJ extends for ∼2× as long as the diffraction-limited focus (Supplement 1, Fig. S10). Thus, the PNJ is able to probe a greater number of fibers in the axial direction while maintaining subdiffraction-limited lateral resolution, increasing sensitivity to SHG polarization anisotropy and, by extension, their changes as a result of drug treatment or disease.

Currently, imaging of such nanoscale changes in collagen is carried out by electron microscopy (EM) [56] and occasionally with atomic force microscopy (AFM) [57]. While higher resolution is possible with these methods, none allow for the simplicity, nor can they match the throughput of optical techniques. Using p-SR-SHG methods demonstrated in this work, significantly high resolution can be achieved without the complexity of sample preparation or sophisticated equipment. Furthermore, in EM, heavy metal stains need to be used to generate contrast [58], while for AFM analysis the collagen needs to be isolated [59] and analysis of native tissue is unrealistic.

## 3. CONCLUSIONS

SR-SHG is able to improve the resolution of a conventional SHG microscope and facilitate label-free SR imaging in a simple and cost-effective way. In this work, we showed both qualitatively and quantitatively that PNJ-assisted SR-SHG can be achieved. We demonstrated a ∼2.3× improvement over diffraction-limited imaging. While imaging in the far field, we show that near-field components of the PNJ contribute to the subdiffraction-limited spot sizes to enable SR-SHG observed with this method. However, we note the important difference of our technique with conventional scanning near-field probes, which is that in our application the probe is not scanned mechanically nor does it capture the near-field feedback or signal, but rather a far-field confocal laser scanning is used to obtain useful sample information. This makes the nanojet much more versatile for imaging in complex soft 3D samples. With the availability of suitable resolution standards for SHG imaging and using smaller spheres to generate PNJs, even higher resolutions could be achieved. The key parameters affecting SR-SHG imaging were characterized and optimized, including the effect of sphere size and distance from the focal plane. We presented a way to increase FOV by using an array of self-assembled spheres. In a significant new finding, we show that despite the tight focusing properties of microspheres, the polarization state is maintained in PNJs, allowing pSR-SHG imaging, wherein polarization anisotropy of SHG signals can be measured. We believe the preservation of polarization in a PNJ will have widespread applications in a number of imaging modalities, including fluorescence to enhance contrast and/or sensitivity to changes. In our case, pSR-SHG improved sensitivity further over both diffraction-limited as well as over normal PNJ-assisted SR-SHG. In another significant result for a highly relevant application, we were able to observe nanoscale changes in collagen disorder in exemplar biological samples (lung tissue spheroid sections) that were invisible to diffraction-limited SHG imaging. Our pSR-SHG technique overcomes the issues of sample preparation and sophisticated instrumentation associated with AFM and EM techniques and is label-free. It can be performed on cells, native tissues (and their sections) directly, making it highly suitable for biomedical research and clinical translation. pSR-SHG can provide unprecedented nanoscale insight into biological harmonophoric-structures such as collagen and thus has the potential to transform our understanding of a wide range of diseases and effects of treatments, including in cancers and fibrosis.

### A. MATERIALS AND METHODS

#### 1. SHG Imaging System

**Fig. 8.**
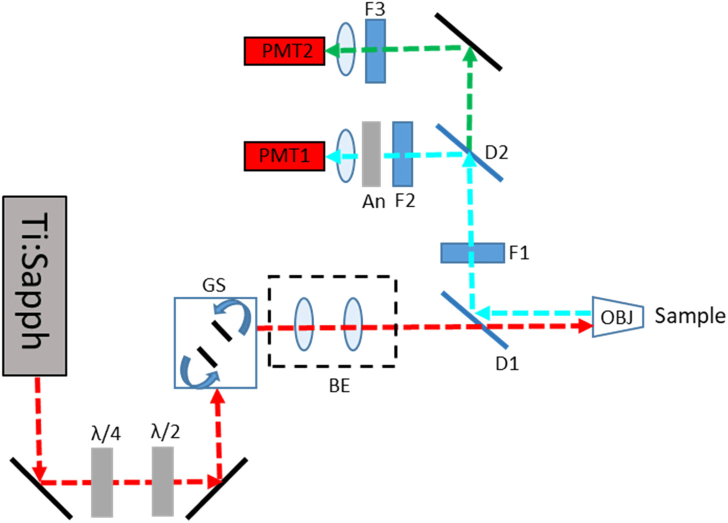
Schematic of the SHG imaging system. The linearly polarized output of a femtosecond-pulsed laser (Ti:Sapph) is passed through a quarter- (λ/4) and half-wave plate (λ/2) to provide precompensation of induced ellipticity and to rotate the linear polarization, respectively. The beam is scanned by a pair of galvanometric mirrors; excitation is separated from emission via a dichroic mirror (D1) and signal cleaned using a short-pass filter (D2). SHG signal is separated using a dichroic mirror (D2) and filtered (F2) before being focused onto PMT1. For polarization-sensitive detection, an analyzer (An) was placed immediately after F2. Longer wavelength signals were detected on PMT 2 after filtering with an appropriate bandpass filter (F3). GS, galvo scanner; BE, beam expander.

SHG imaging was performed on a home-built multiphoton imaging system (Fig. 8) consisting of a femtosecond-pulsed laser (MaiTai, Spectra Physics) that generates 120 fs pulses with a 80 MHz repetition rate, coupled into a Leica DMRB upright microscope stand. Laser scanning is performed using a pair of galvanometric mirrors (Cambridge Instruments).The beam is expanded using a telescope to overfill the objective back aperture (Leica 63×, 1.2 NA, water immersion) and is directed into the objective using a dichroic mirror (Semrock FF685-DI02-25X36). The signal is collected in the backwards direction and separated from the excitation laser by the same dichroic mirror, then cleaned using a short-pass filter with a 694 nm cutoff. SHG was excited at 800 nm for all nonpolarization-based images and at 808 nm for all polarization-based images. SHG was separated from other signals using a dichroic mirror (Semrock, FF458-Di02) and was detected afterward through a bandpass filter centered at 400±20nm (Thorlabs, FB400-40). Detection was performed by focusing onto a PMT (Hamamatsu, H10722-01). For polarization anisotropy measurements, an analyzer (Thorlabs, LPVISE100-A) was placed directly in front of the PMT. The excitation polarization was controlled using a combination of a half-wave plate (Thorlabs, WPH10M-808) to rotate the linearly polarized output of the laser and a quarter-wave plate (Thorlabs, zero order 808 nm) to precompensate for ellipticity induced by the scanning mirrors and excitation dichroic. The polarization state of the fundamental beam at the sample was determined using a linear polarizer (analyzer) and power meter. For a given orientation of the analyzer, the extinction ratio was maximized at the linear polarization of the fundamental deemed to be orthogonal to the analyzer transmission axis.

#### 2. PNJ Generation for Imaging

Spheres made of barium titanate glass (BTG), (refractive index of 1.9) were used. Their high refractive index contrast allowed them to be used with water (n=1.33) to form a PNJ in water immersion medium with corresponding objectives commonly used for biological and multiphoton applications. Amorphous $BaTiO_{3}$ microspheres were purchased from Cospheric (Santa Barbara, CA, U.S.) in two size ranges of 5–22 µm and 53–63 µm. Spheres were suspended in water by rapid pipetting and a drop of the water deposited onto the sample surface. Microspheres rapidly sunk to the sample surface, and the water droplet formed the immersion medium for imaging. Self-assembled sphere arrays were formed by allowing the droplet of sphere suspension to dry where surface tension acts to pull the spheres into an array. A droplet of clean water (not containing spheres) was then gently deposited on top of the array to act as the immersion medium.

#### 3. Barium Titanate Sample Preparation

Barium titanate nanocrystals <100nm specified size (Sigma-Aldrich) were suspended in ethanol, ultrasonicated for 10 min to break up aggregates, and then a 10 µL drop was placed onto a clean microscope slide and left to air dry.

#### 4. Mouse Tendon Sample Preparation

All mouse experiments were carried out in accordance with the Animals (Scientific Procedures) Act 1986 set out by the UK Home Office. Female C57BL/6 mice aged between 4 and 6 months were culled via ${CO}_{2}$ and cervical dislocation. The tail was removed using scissors and the tendon removed using a pair of forceps and was fixed in 4% paraformaldehyde for 6–8 h. They were then washed in phosphate buffered saline (PBS) immediately before being mounted on a microscope slide and sealed under a coverslip (#1.5; 0.17 mm thick) before imaging.

#### 5. Lung Tissue

Dewaxed formalin-fixed paraffin-embedded human lung tissue sections (5 µm) were from tissue surplus to clinical requirements from lung biopsies of patients with a subsequent diagnosis of idiopathic pulmonary fibrosis (IPF) under the approval of the Southampton and South West Hampshire and the Mid and South Buckinghamshire Local Research Ethics Committees (Ref. 07/H0607/73). All subjects gave written informed consent.

#### 6. Spheroid Sample Preparation

Details of spheroid growth have been described in detail previously [53]. Briefly: Primary lung fibroblasts from a patient with IPF were seeded in Transwell inserts under normal conditions for 24 h before the media were replaced with that containing BAPN or control conditions. After 6 weeks of growth under these conditions, the spheroids were harvested, fixed in 4% paraformaldehyde, paraffin-embedded, and 5 µm sections cut ready for imaging.

#### 7. TEM

As previously described [53], for TEM, spheroids were fixed in 3% glutaraldehyde in 0.1M caco-dylate buffer at pH 7. Spheroids were postfixed sequentially in osmium/ferocyanide fixative thiocarbohydrazide solution, osmium tetroxide, uranyle acetate, and Walton’s lead aspartate solution before dehydration in graded ethanol and acetonitrile. Samples were embedded in Spurr resin and 100 nm ultrathin sections were visualized using an FEI Tecnai 12 transmission electron microscope (FEI Company, Hillsboro, OR, U.S.).

#### 8. SHG Resolution Standard Fabrication

Silicon membranes were commercially sourced (Norcada, Inc.) (200 nm thick polycrystalline silicon membrane 1×1mm, 200 µm silicon frame 5×5mm). Focused ion beam (FIB) milling was performed on an FEI Helios NanoLab 600 FIB system. Each cut was formed by using an ion beam of gallium ions, with an acceleration voltage of 30 keV and beam current of 28 pA.

#### 9. Simulations

Numerical simulations were carried out using COMSOL Multiphysics 5.4, a finite-element method (FEM)-based numerical software package to investigate light propagation through the barium titanate microsphere (refractive index was assumed to be nms=1.9). We adopted full-field formalism with periodic boundary conditions (Floquet) applied on all the four sides of the computational geometry, assuming an infinite 2D periodic structure in the x−y plane, solving the wave equation in a 3D model. The incident beam was assumed to be a plane wave, linearly polarized along x axis propagating from the front side of the microsphere, i.e., along the z direction, and the excitation wavelength was set at 800 nm. The size of the microsphere was taken to be 7 µm and was positioned at the center of the computational window and also assumed to be embedded in water medium (n=1.33). The relative permeability was taken to be µr=1. The entire structure was divided into domains and subdomains, and each domain was meshed using free tetrahedral meshing of maximum element size 6 elements per wavelength outside the microsphere and extremely fine meshing within the sphere. Direct solver method was adopted to solve for the wave equation.

#### 10. Calculation of Polarization Anisotropy

Polarization anisotropy images were created using a home written Fiji macro in which the key steps are as follows: A difference image was generated by ($I_{par}-I_{perp}$); this was divided by a total intensity image ($I_{par}+2I_{perp}$). The total intensity image was used to select the region of the image containing a signal and for display, all pixels outside of this region were set to 0. Only the regions containing a signal were used for further anisotropy analysis.

#### 11. Collagen Fiber Analysis

Collagen fiber analysis was performed using CT-FIRE V1.3. SHG intensity images were converted to 8-bit format before being batch-processed using default parameters. Four parameters were measured to describe fiber morphology: width, length, straightness, and angle.

## Data Availability

Data underlying the results presented in this paper are not publicly available at this time but may be obtained from the authors upon reasonable request.

## Supplemental document

See Supplement 1 for supporting content.
