# Supplementary material for: Superresolved polarization-enhanced second-harmonic generation for direct imaging of nanoscale changes in collagen architecture
Source: Optica. 2021 May 13;8(5):674–85. doi: 10.1364/OPTICA.411325 (PMC8237832; doi:10.1364/OPTICA.411325)
Supplement: Supplementary file 1 [file optica-8-5-674-s001.pdf]

Supplemental Document

optica

Superresolved polarization-enhanced second-harmonic generation for direct imaging of nanoscale changes in collagen architecture: supplement

PETER B. JOHNSON,[1,2] ARTEMIOS KARVOUNIS,[3] H. JOHNSON SINGH,[4] CHRISTOPHER J. BRERETON,[5] KONSTANTINOS N. BOURDAKOS,[1,2] KERRY LUNN,[6] JAMES J. W. ROBERTS,[6] DONNA E. DAVIES,[2,5] OTTO L. MUSKENS,[4] MARK G. JONES,[2,5] AND SUMEET MAHAJAN[1,2,*]

[1] *School of Chemistry, Faculty of Engineering and Physical Sciences, University of Southampton, Southampton, UK*
[2] *Institute for Life Sciences, University of Southampton, Southampton, UK*
[3] *Optoelectronics Research Centre and Centre for Photonic Metamaterials, University of Southampton, Southampton, UK*
[4] *Physics and Astronomy, Faculty of Engineering and Physical Sciences, University of Southampton, Southampton, UK*
[5] *NIHR Southampton Biomedical Research Centre, University Hospitals Southampton, Clinical and Experimental Sciences, Faculty of Medicine, University of Southampton, Southampton, UK*
[6] *Synairgen Research Ltd., Southampton, UK*
[*] *Corresponding author: s.mahajan@soton.ac.uk*

This supplement published with The Optical Society on 13 May 2021 by The Authors under the terms of the Creative Commons Attribution 4.0 License in the format provided by the authors and unedited. Further distribution of this work must maintain attribution to the author(s) and the published article's title, journal citation, and DOI.

Supplement DOI: https://doi.org/10.6084/m9.figshare.14230049

Parent Article DOI: https://doi.org/10.1364/OPTICA.411325

Supporting Information

Super-resolved polarization-enhanced second harmonic generation for direct imaging of nanoscale changes in collagen architecture

Peter B. Johnson[1,5], Artemios Karvounis[2], H. Johnson Singh[3], Christopher J. Brereton[4], Konstantinos N. Bourdakos[1,5], Kerry Lunn[6], James JW Roberts[6], Donna E Davies[4,5] , Otto L. Muskens[3], Mark G Jones[4, 5], Sumeet Mahajan[1,5]

1: School of Chemistry, Faculty of Engineering and Physical Sciences, University of Southampton, Southampton, UK

2: Optoelectronics Research Centre and Centre for Photonic Metamaterials, University of Southampton, Southampton, UK

3: Physics and Astronomy, Faculty of Engineering and Physical Sciences, University of Southampton, Southampton, UK

4: NIHR Southampton Biomedical Research Centre, University Hospitals Southampton, Clinical and Experimental Sciences, Faculty of Medicine, University of Southampton, Southampton, UK

5: Institute for Life Sciences, University of Southampton, Southampton, UK

6: Synairgen Research Ltd, Southampton, UK

1. Confirmation of SHG signals excited via photonic nanojet and resolution improvement with increased power

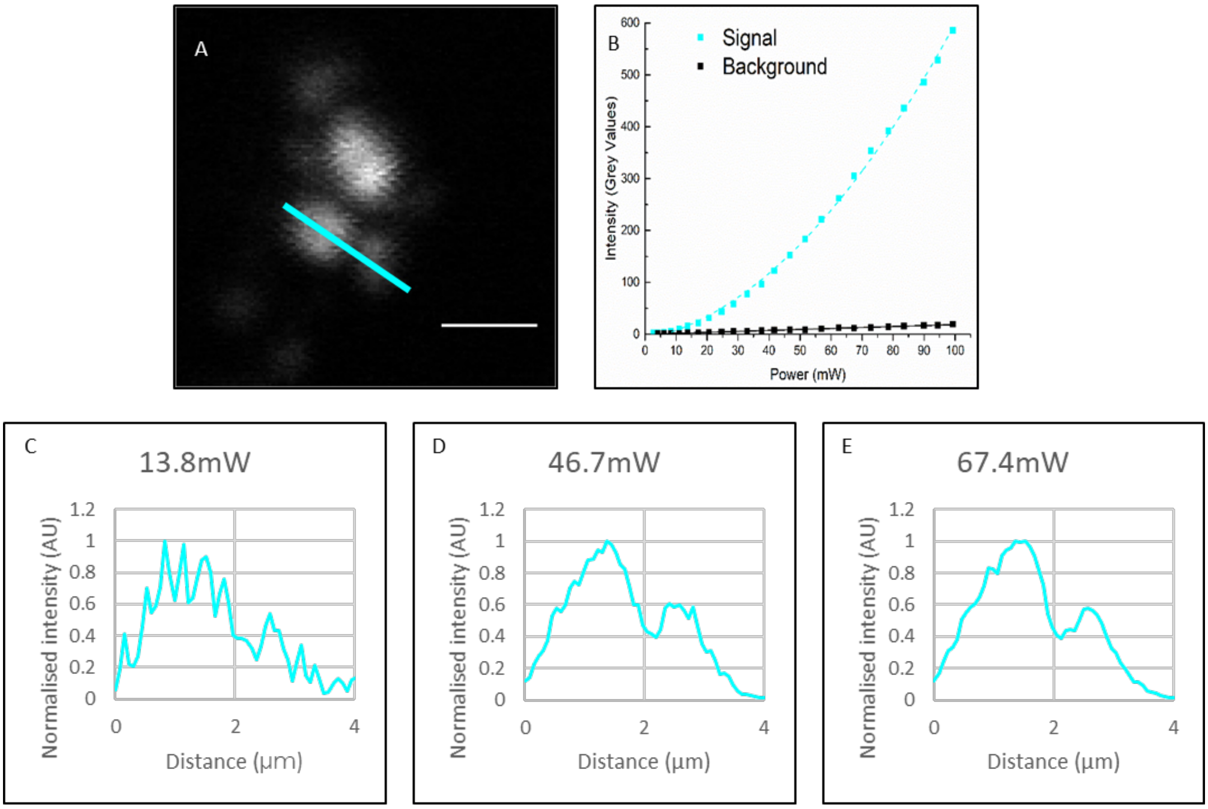

Figure S1: Characterisation of the SHG response when using PNJs. **A** an example image of Barium Titanate nanocrystals imaged through a microsphere at increasing incident powers. Scale bar 2µm. **B** Plots of signal and background against incident power, the signal shows a quadratic relationship to power whereas the background increases linearly confirming the signal as SHG. **C,D,E** Intensity plots taken across the cyan line marked in **A** as power is increased **(C→E)** the signal to noise ratio increases and the peaks become more easily resolved. Increasing the power improves resolution.

2. Improvement in resolution on SHG-active standard samples

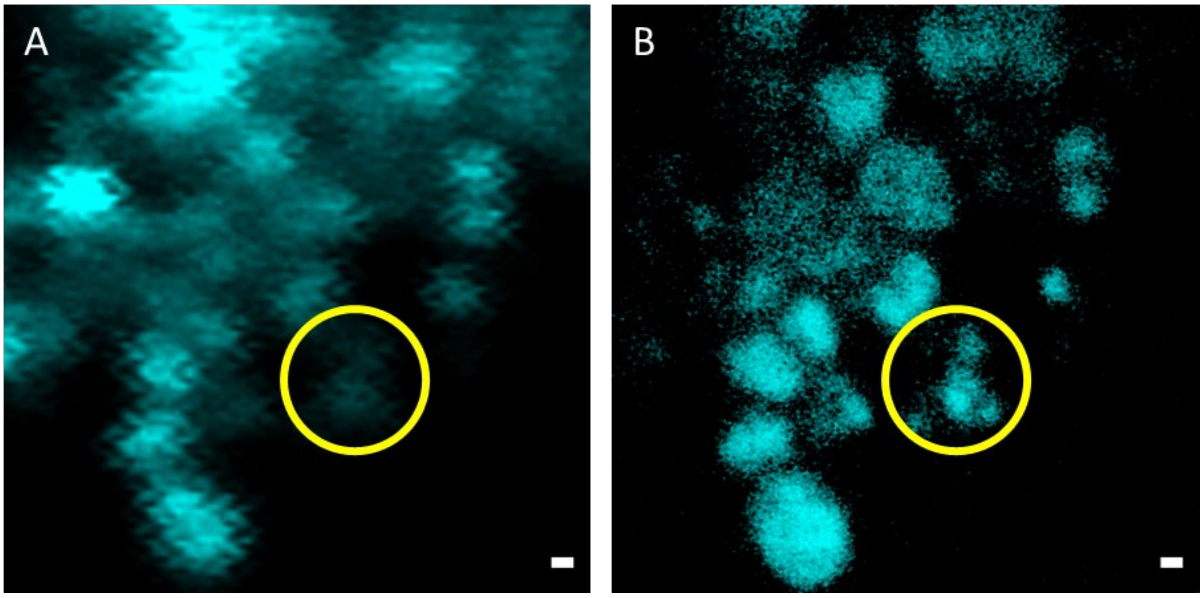

Figure S2. PNJs improve the resolution of a Second Harmonic microscope. **A:** A diffraction-limited SHG image of $BaTiO_3$ nanocrystals and **B** an SR-SHG image of the same location. Scale Bar 500 nm.

3. Verification of presence/abundance of collagen in tissue samples investigated in this study

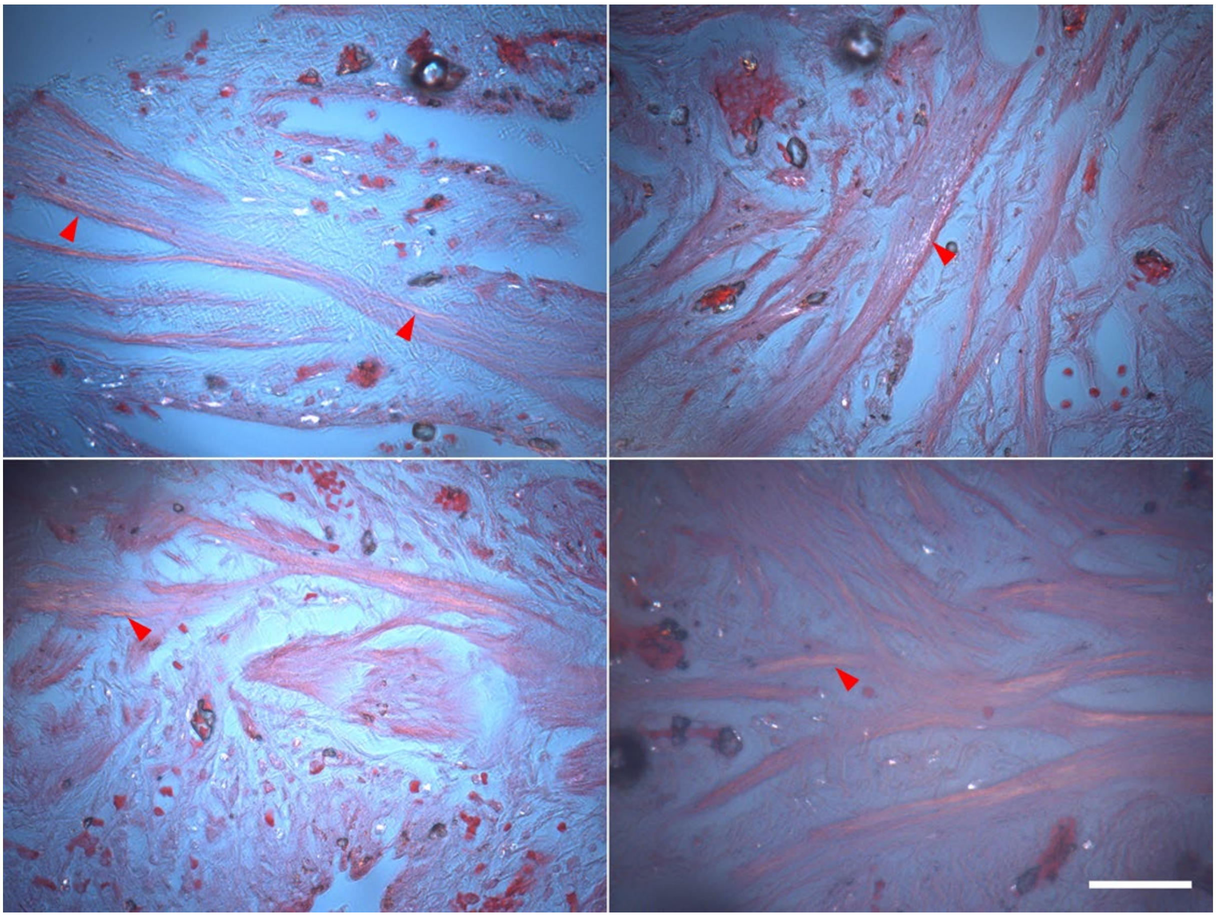

Figure S3 Confirmation of collagen abundance in the tissue samples studied. Example images taken of the same lung tissue sample (with a parallel section) showing the distribution of collagen stained with PicroSirius Red and imaged using polarised light microscopy. Collagen fibres appear as gold/white on a pink and blue background, red arrows indicate areas of high collagen concentration Scale bar 50μm.

4. Optimization of parameters for PNJ-assisted SR-SHG

Previous studies have shown that the degree of magnification and the improvement in resolution generated by a microsphere are dependent on the sphere size [45] and the relative z position [46] of the objective focus and the sphere-sample contact. To characterise this for SHG imaging a patterned sample of known dimensions was generated from a 200 nm thick silicon film. A series of slits were cut into the film using focussed ion beam (FIB) milling, the interface between the silicon and the immersion water that filled the slit provided the lack of inversion symmetry required to be SHG active. PNJ behaviour consistent with previously published results [9,47] was observed. Shorter and narrower PNJs are formed by smaller spheres [48] [6] however, they have a focal range closer to the sphere-sample contact. For thicker samples, out of focus excitation not contributing to the PNJ creates a strong background over which the signal is not observable. Therefore, the relationship between imaging range and sample thickness must be considered when selecting a sphere size to use. Sphere size also affects the achievable resolution in SR-SHG with smaller spheres providing better resolution due to the smaller photonic nanojet [6] but with a smaller field of view. Based on all these considerations, and given that tighter PNJs are expected of smaller spheres we chose 14 µm spheres at the z-position z+20µm for highest magnification for establishing the limit of resolution of SR-SHG while for tissue imaging we chose 60 µm spheres to increase FOV whilst maintaining subdiffraction-limited resolution.

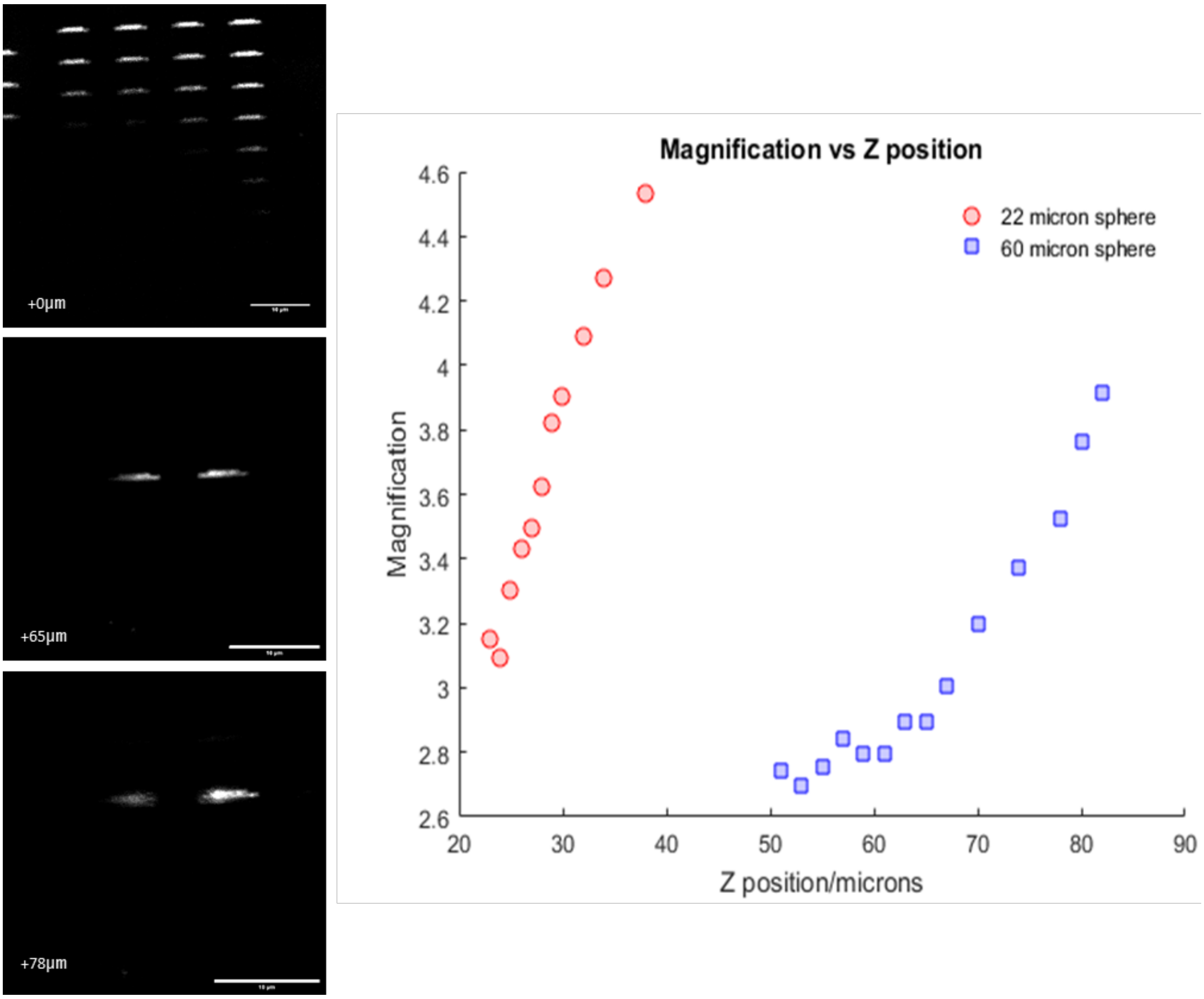

Figure S4 Magnification dependence with PNJs. The degree of magnification by microspheres depends on the relative position of the objective focal plane and the sphere sample contact (Z position) and the size of the sphere. 3 example images taken through a 60µm sphere. The top image is taken focussed at the sample surface, the sample contains a grid of 4 x 8 slit features, and the dark region indicates the location of the sphere. The images taken at z+65 µm and z+78 µm show that the image generated through the sphere increases in magnification

as z increases. The z+78 µm image also shows that the image starts to become defocussed as z increases past the focal range, Images were taken through microspheres of 2 different sizes, 22 µm and 60 µm, The z-ranges for obtaining magnified images were 50-85 µm for the larger spheres and 20-40 µm for the smaller spheres. The graph shows that smaller size spheres produce a greater magnification than the larger spheres but over a narrower focal range. The focal range of smaller spheres occurs closer to the sphere than that of larger spheres. Scale bar 10 µm.

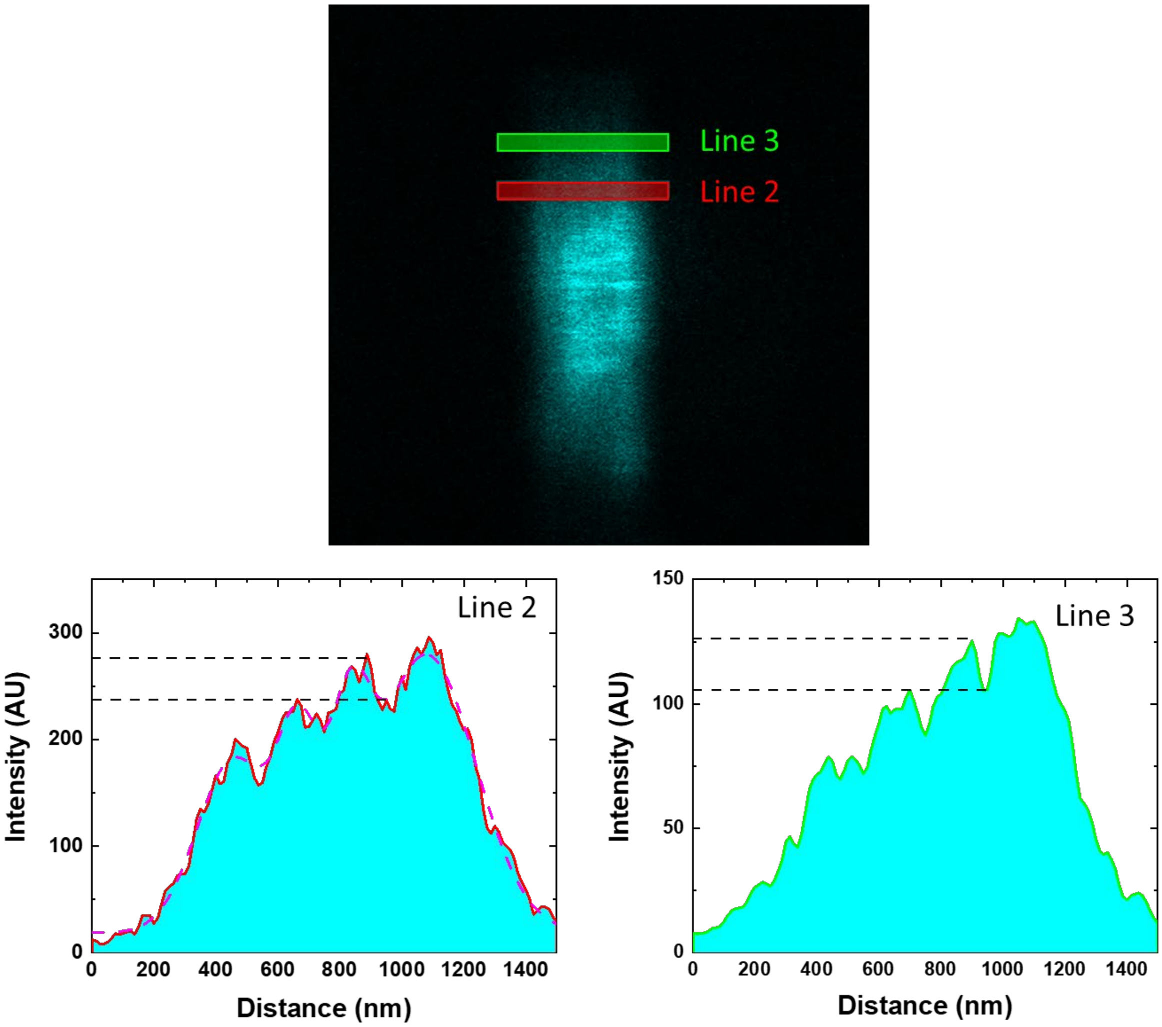

Figure S5: Verification of PNJ performance. Additional intensity plots of the silicon slit sample.

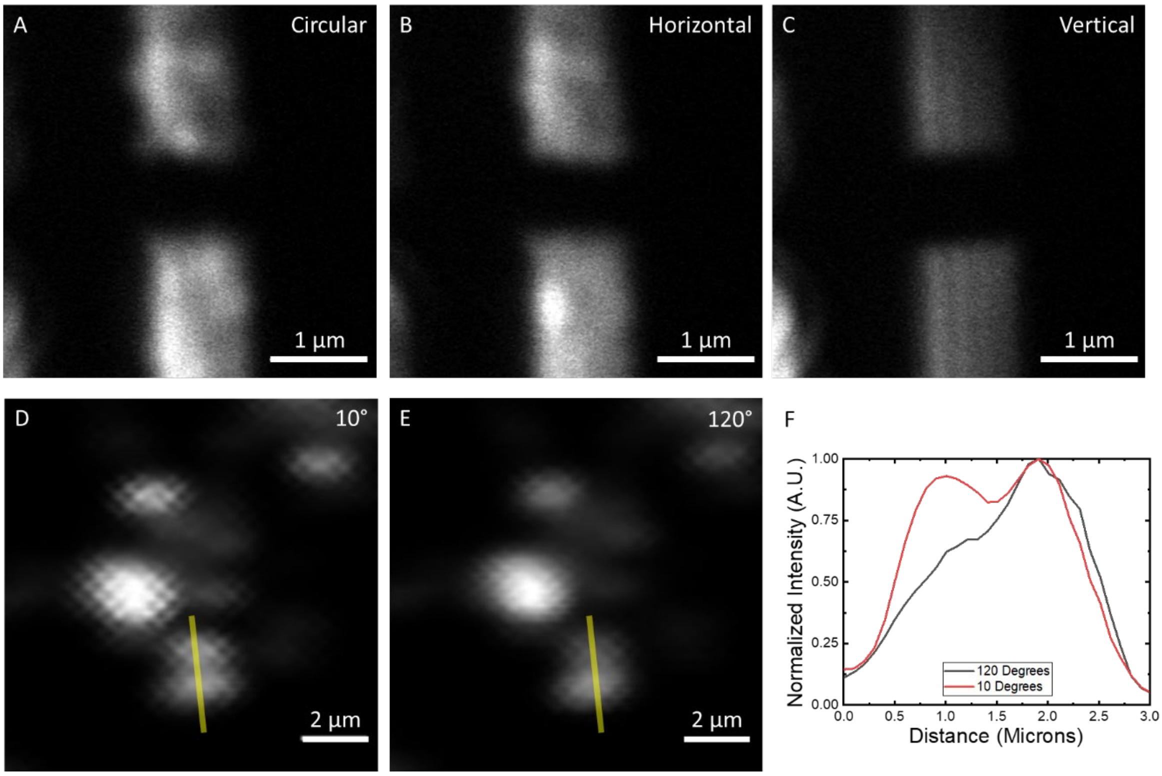

Figure S6 The effects of polarization on resolution. Top row: When the excitation field is circularly polarized **(A)** or polarized perpendicular to the long axis of the slit **(B)** the slits cannot be resolved. When the excitation is polarized parallel to the slits **(C)** the resolution is improved and the slits can be resolved. Bottom Row: SHG intensity images of $BaTiO_3$ nanocrystals with linear excitation polarization orientated at 10° (D) and 120° (E) to horizontal. (F) Intensity plots taken along the yellow lines in D/E, depending on the polarization the neighbouring crystals are either resolved or not.

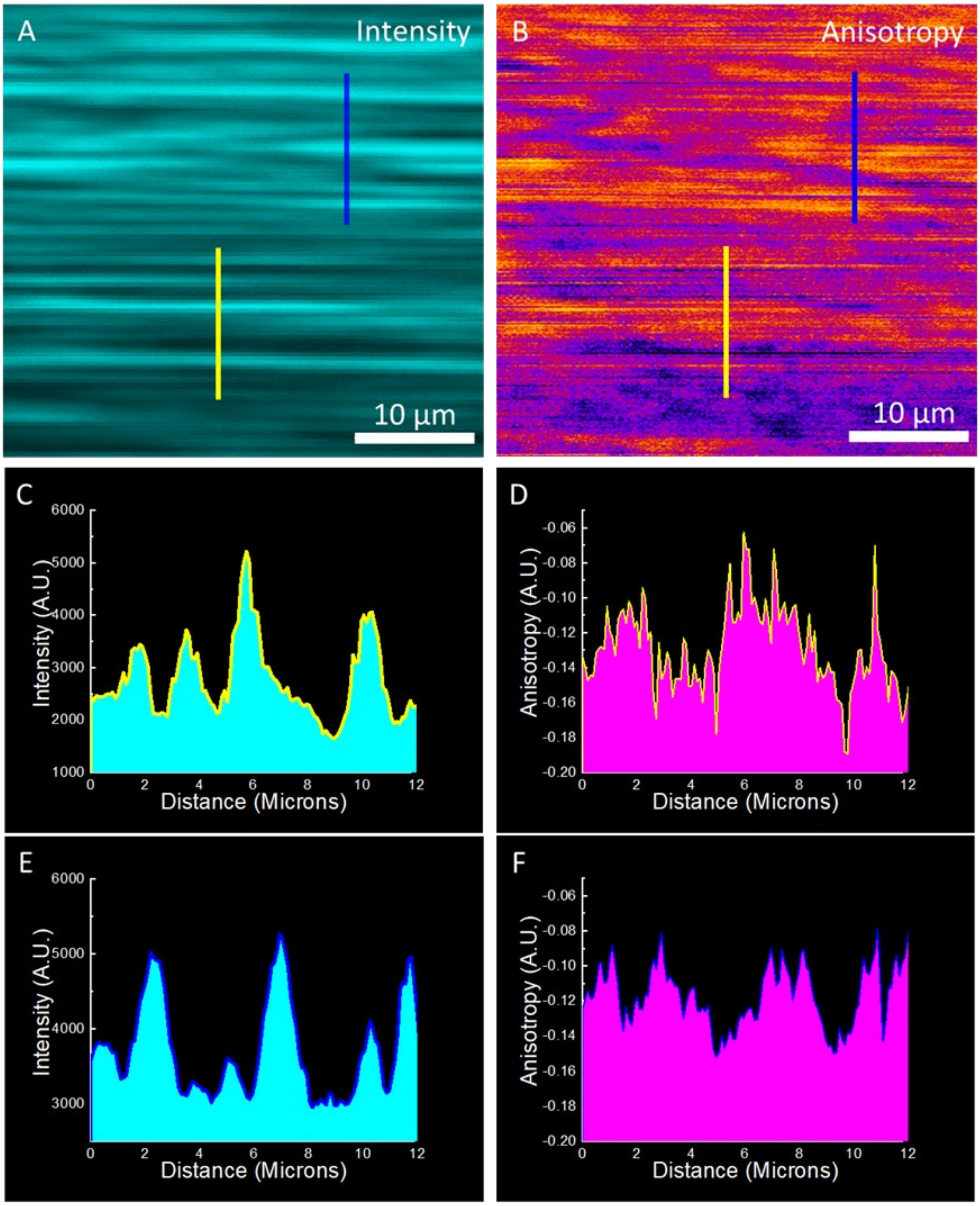

Figure S7: Polarization anisotropy imaging Mouse tail tendon with defined fibre structure displayed using the SHG intensity (A) and using the polarization anisotropy (B). Intensity plots of the yellow (C) and blue (D) lines in (A) show clear defined fibre features. The corresponding Anisotropy measurements taken from the yellow and blue line in (B) (D and F respectively) reveal features of finer frequency than the intensity

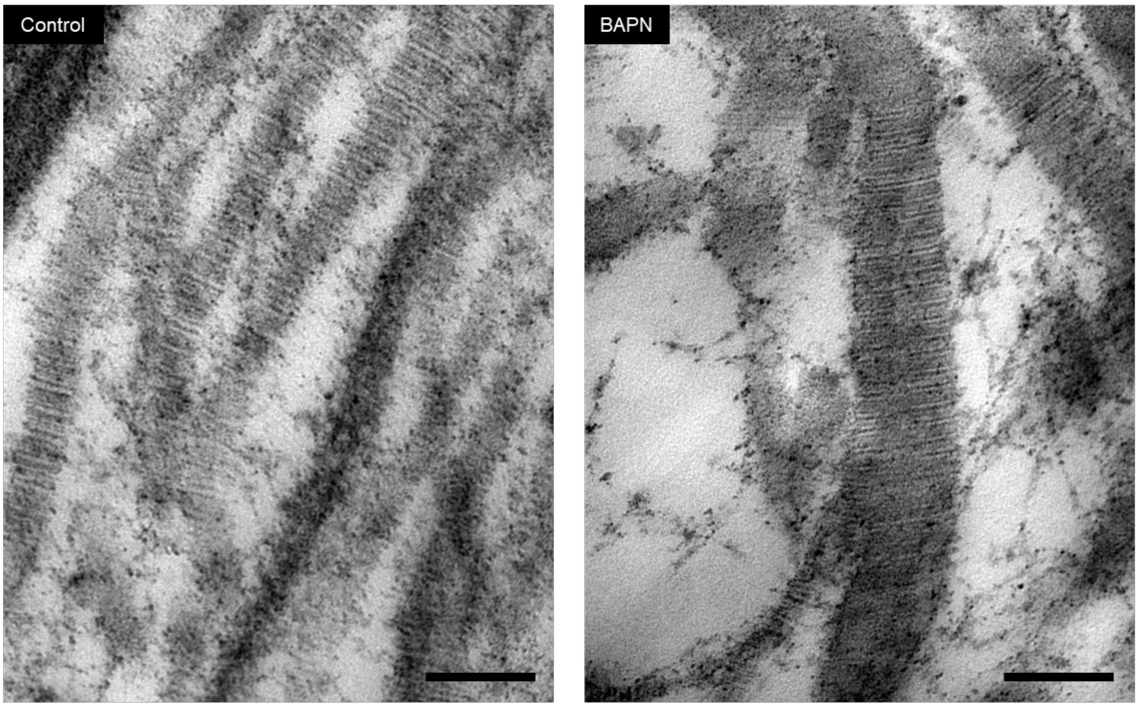

Figure S8: TEM verification of changes in collagen architecture on treatment with BAPN. IPF fibroblasts were grown for 6 weeks in the 3D in vitro model of fibrosis with vehicle control, or 1 mM BAPN, as indicated. Transmission electron microscopy images of collagen fibrils in longitudinal cross-section identifies that with BAPN there is an increase in fibril diameter and a marked dysregulation of fibril structure including irregular profiles. Scale bar 100 nm.

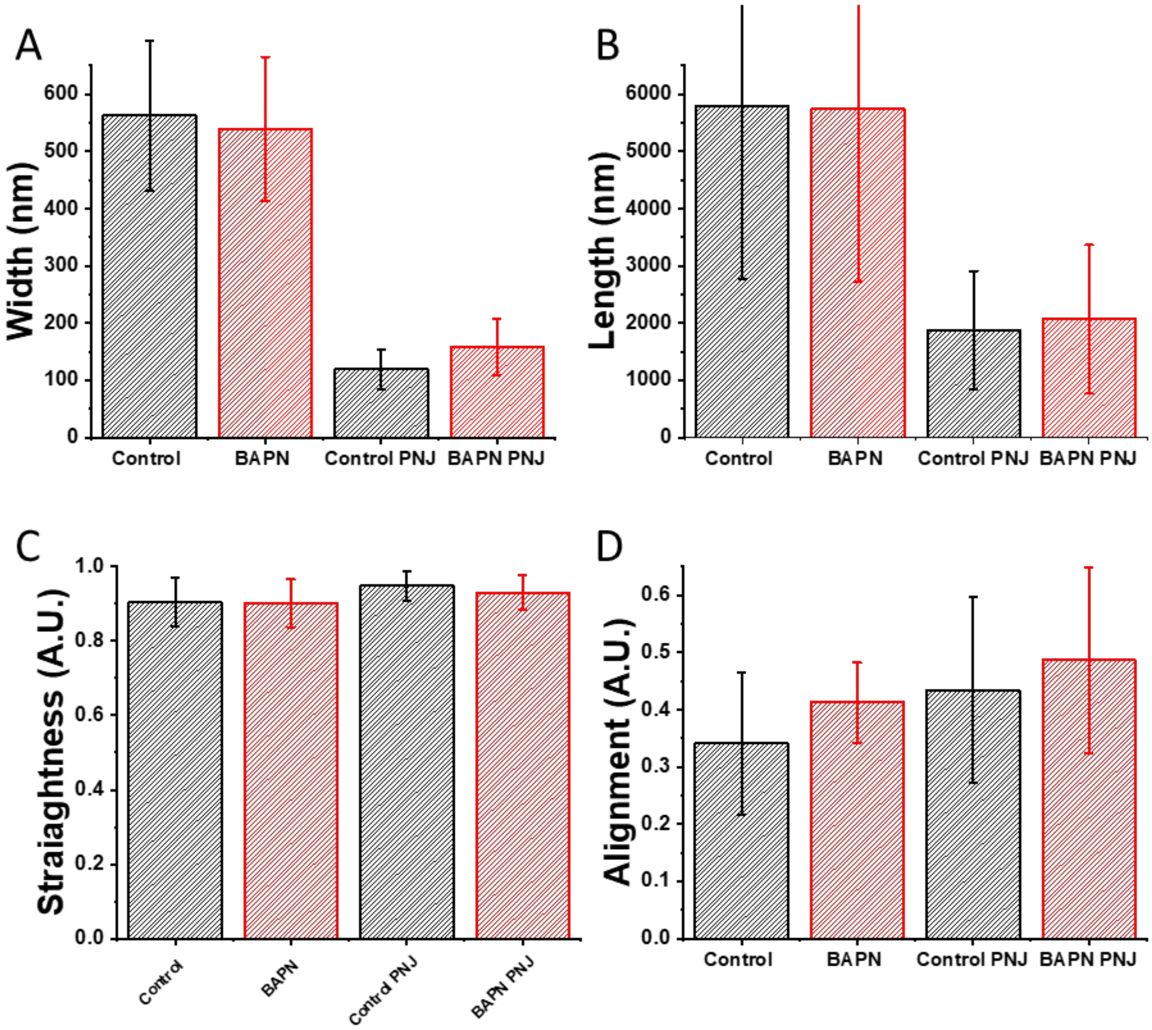

Figure S9: Conventional analysis of collagen fibres. Image analysis of fibre parameters. A. Analysis of fibre width, Diffraction-limited imaging showed no difference between Control and BAPN treat samples. PNJ imaging showed a slight increase in fibre width in BAPN treated (158 nm) vs control (119 nm) although this was not statistically significant. PNJ analysis indicated many narrower fibres than diffraction-limited imaging. B Analysis of fibre length. Diffraction-limited imaging showed no significant differences between Control and BAPN treated spheres, this was also true when imaged using PNJs. PNJ imaging indicated shorter fibres in both cases than diffraction-limited imaging. C/D Analysis of fibre straightness (C) and alignment (D) revealed no significant differences when analysed via diffraction-limited or PNJ assisted imaging.. CT-FIRE was used for the analysis. Four parameters were measured to describe fibre morphology, width, length, straightness and alignment, for each of these parameters there is no significant distance between control and BAPN treated case. A total of 15 FOVs were tested for Control and BAPN diffraction-limited case and 20 FOVs for PNJ assisted images. Error bars = 1 standard deviation from the mean

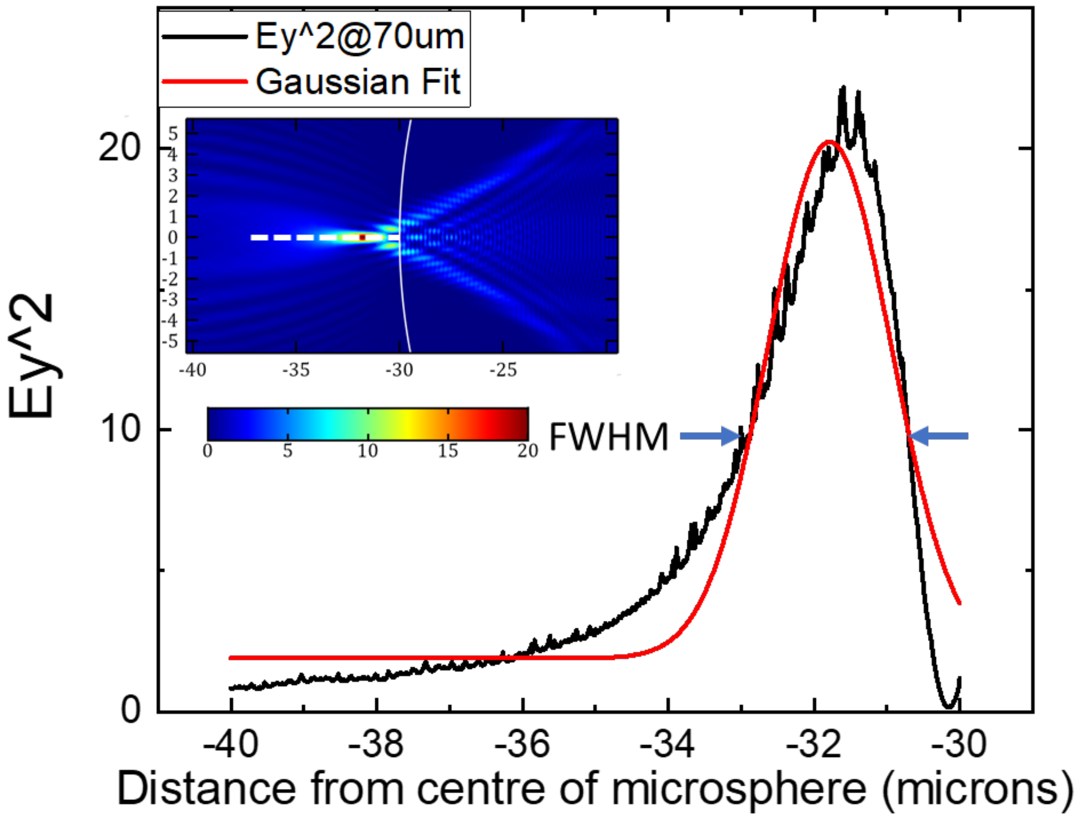

Figure S10: Axial extent of the photonic nanojet. Inset: Simulated intensity distribution of a 60 µm diameter microsphere (n = 1.9) immersed in water (n = 1.33), illuminated by a beam of 800 nm wavelength. The beam is focussed by a 1.2 NA objective and the objective focus in the absence of the microsphere is 70 µm to the shadow side (left hand side) of the left hand surface of the sphere. An intensity plot taken along the white dashed line in the inset give the black line. Gaussian fitting of the black line gives a FWHM of 1.99 µm, as SHG is a multiphoton process the SHG generating distribution is expected to be narrower by a factor of √2 giving a FWHM of 1.4 µm.. The diffraction-limited focus as calculated from [55]is 0.657 µm.
